# The learning curves of a validated virtual reality hip arthroscopy simulator

**DOI:** 10.1007/s00402-020-03352-3

**Published:** 2020-01-27

**Authors:** Jonathan D. Bartlett, John E. Lawrence, Matthew Yan, Borna Guevel, Max E. Stewart, Emmanuel Audenaert, Vikas Khanduja

**Affiliations:** 1 School of Clinical Medicine, Addenbrooke’s Hospital, Cambridge, UK; 2 Department of Trauma and Orthopaedics, Ghent University, Ghent, Belgium; 3 Young Adult Hip Service, Department of Trauma and Orthopaedics, Addenbrooke’s, Cambridge University Hospitals NHS Foundation Trust, Addenbrooke’s Hospital, Box 37, Hills Road, Cambridge, CB2 0QQ UK

**Keywords:** Virtual reality, Hip arthroscopy, Simulator, Learning curve, Training effect, Scope manipulation, Arthroscope

## Abstract

**Introduction:**

Decreases in trainees’ working hours, coupled with evidence of worse outcomes when hip arthroscopies are performed by inexperienced surgeons, mandate an additional means of training. Though virtual reality simulation has been adopted by other surgical specialities, its slow uptake in arthroscopic training is due to a lack of evidence as to its benefits. These benefits can be demonstrated through learning curves associated with simulator training—with practice reflecting increases in validated performance metrics.

**Methods:**

Twenty-five medical students with no previous experience of hip arthroscopy completed seven weekly simulated arthroscopies of a healthy virtual hip joint using a 70° arthroscope in the supine position. Twelve targets were visualised within the central compartment, six via the anterior portal, three via the anterolateral portal and three via the posterolateral portal. Task duration, number of collisions (bone and soft-tissue), and distance travelled by arthroscope were measured by the simulator for every session of each student.

**Results:**

Learning curves were demonstrated by the students, with improvements in time taken, number of collisions (bone and soft-tissue), collision length and efficiency of movement (all *p* < 0.01)*.* Improvements in time taken, efficiency of movement and number of collisions with soft-tissue were first seen in session 3 and improvements in all other parameters were seen in session 4. No differences were found after session 5 for time taken and length of soft-tissue collision. No differences in number of collisions (bone and soft-tissue), length of collisions with bone, and efficiency of movement were found after session 6.

**Conclusions:**

The results of this study demonstrate learning curves for a hip arthroscopy simulator, with significant improvements seen after three sessions. All performance metrics were found to improved, demonstrating sufficient visuo-haptic consistency within the virtual environment, enabling individuals to develop basic arthroscopic skills.

## Introduction

The increasing utilisation of more technically demanding surgical procedures, coupled with decreases in trainees’ caseloads and working hours, has led to difficult learning curves in orthopaedic surgical training [1–7]. This is particularly true for the rapidly expanding field of hip arthroscopy. The geometry of the joint, combined with its thick soft-tissue envelope, makes hip arthroscopy particularly challenging and long learning curves have previously been described [3, 8–10]. Concerns regarding patient safety, fuelled by evidence of worse outcomes when arthroscopic procedures are performed by inexperienced surgeons, mandate the development of an additional means of training arthroscopic surgeons [3, 9].

Though virtual reality (VR) simulation training has been increasingly adopted by other surgical specialities, its use in arthroscopic surgical training remains limited [5, 11]. This slow uptake is, in part, the result of a lack of robust evidence as to the benefits of VR training to both the trainee and the patient [5, 11]. One means of demonstrating these benefits to the trainee is through the analysis of the learning curves associated with simulator training—with repeated practice reflecting measurable increases in validated performance metrics. Though virtual reality simulators have been suggested to shorten surgical learning curves, there are currently only reports in the literature on box simulators and low fidelity simulators [11–13]. Without demonstration of learning curves, it cannot be assumed that the visuo-haptic feedback provided by the VR simulator is sufficiently consistent to allow skill development.

The purpose of this study was to investigate how novice performance on a VR hip arthroscopy simulator’s visualisation module changed with repeated practice. We hypothesised that repeated use of the simulator would lead to significant decreases in time taken, number of collisions and distance travelled by the arthroscope before reaching a plateau, thus demonstrating learning curves.

## Methods

### Participants

Twenty-five medical students were voluntarily recruited for this study. Basic demographic information including gender, age, year of study and arthroscopic experience was collected.

### Simulator

**Fig. 1:**
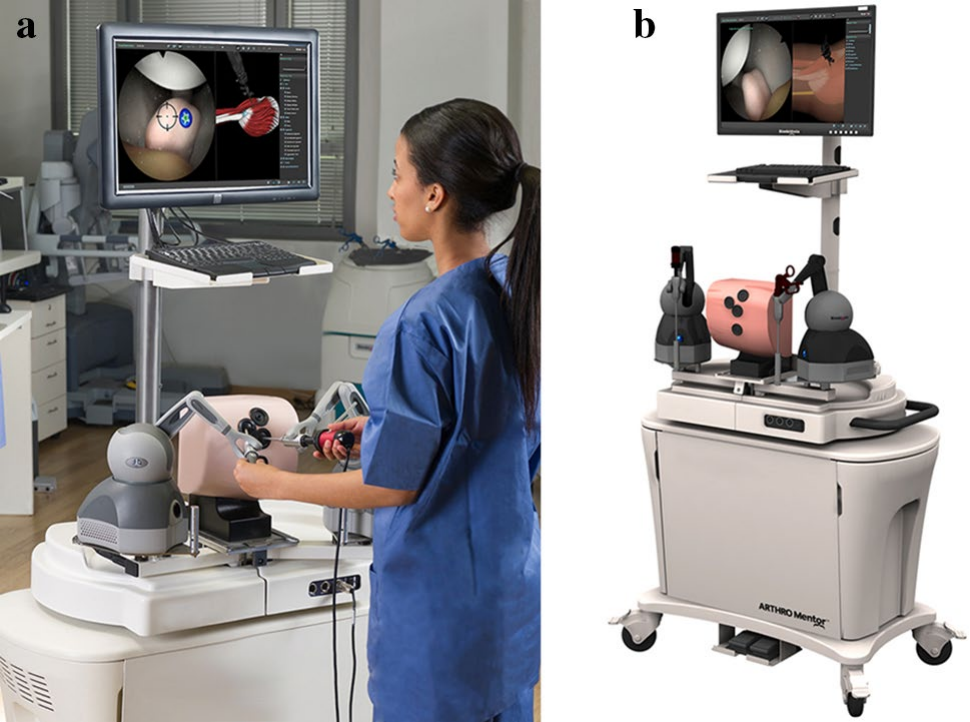
**a** and **b** User interface of the Simbionix Arthro Mentor, consisting of a computer with monitor, a mannequin, and two haptic feedback devices that provide tactile feedback to a pair of instruments via connecting motors

The previously validated Simbionix ArthroMentorVR simulator (3D Systems, Littleton, USA) was used in this study [14, 15]. This simulator consisted of a computer and monitor, a mannequin, and two haptic feedback devices capable of providing tactile feedback to a pair of instruments via connecting motors (Fig. 1a, b). The mannequin had four predefined 5 mm arthroscopy portals at the modified anterior, anterior, anterolateral, and posterolateral sites. The images of the virtual joint were displayed on the monitor in response to the movements of the operator.

### Arthroscopy simulator protocol

All participants received an identical standardised introduction by the same individual. In this, participants were introduced to the basic principles of hip arthroscopy and VR simulation and shown a demonstration of the full diagnostic examination of the hip joint on the simulator. Following this, each participant was given a familiarisation period of exactly 3 min in which they could examine the hip joint from all three portals.

Each participant completed seven identical diagnostic hip examination modules in the supine position. Each participant completed one session every 7 days. Each module began with standardised written instructions on screen, with the procedure starting upon insertion of the arthroscope into the anterolateral portal. The module involved locating a series of 12 consecutive targets within the hip joint using an arthroscope. Six targets were visualized via the anterolateral portal, followed by three via the anterior, and finally three via the posterolateral (Table 1). The name of each target was displayed to the participant on-screen and the order in which the targets appeared was identical for each participant. Participants were required to place each target in the centre of the monitor for 3 s before the name of the next target in the examination sequence was displayed to them on-screen.

**Table 1:** List of targets visualised during diagnostic hip module

Portal site	Targets to be visualised during task
Anterolateral	Posterior transverse ligament Posterior labrum Anterior triangle Anterior labrum Posterior capsule Femoral head
Anterior	Ligamentum teres Posterior transverse ligament Anterior transverse ligament
Posterolateral	Weight-bearing acetabulum Posterior superior labrum Femoral head

During every attempt, the participants’ performance was evaluated by the simulator via a set of predefined metrics. These included the total time taken to complete the procedure, the number of collisions between the soft-tissue and the arthroscope, the number of collisions between bone and the arthroscope, the total time of soft-tissue collisions (seconds), the total length of femoral head scratches (mm), and the distance travelled by the arthroscope (cm).

### Ethical approval

As per the National Health Service (NHS) Health Research Authority’s guidance, this study did not require approval from an NHS Research Ethics Committee [16]. This study was conducted in agreement with the ethical standards of the University of Cambridge, the NHS Research Ethics Committee and the 1964 Helsinki Declaration.

### Statistical analysis

Statistical analysis was performed with version 3.2 of R (Foundation for Statistical Computing, Vienna, Austria). A power analysis was performed to estimate the sample size required to detect improvement across the sessions. On the basis of pilot attempts by the authors, the total task time was estimated to be 900 s for the first session. On the basis of previous studies, the standard deviation for a simulated arthroscopic task was estimated to be 40% of the task time, with a difference of 25% considered clinically relevant [13, 17]. Therefore, to achieve an 80% power (alpha = 0.05), the desired sample size was calculated to be 25.

Demographic data are presented as mean ± standard deviation. A Kilmogorov–Smirnov test revealed the data collected by the simulator to be non-parametric. As such, non-parametric tests were used, and all data presented as median ± interquartile range. A Friedman test with multiple comparison was used to analyse the differences between successive attempts at the module. Comparisons were made between session 1 and subsequent session for all metrics to define the first significant improvement. Comparisons were also made between session 7 and all session to elucidate when individuals’ improvements ceased to be significant.

## Results

The mean age of the students was 21.7 ± 1.8 and the male to female ratio was 19:6. 12% of students were in their third year of study, 48% were in their fourth year of study and 40% were in their fifth year of study. The mean number of times using an arthroscope (simulated or real) amongst the students was 0.3 ± 0.7 and no student had ever used a hip arthroscope (simulated or real).

**Fig. 2:**
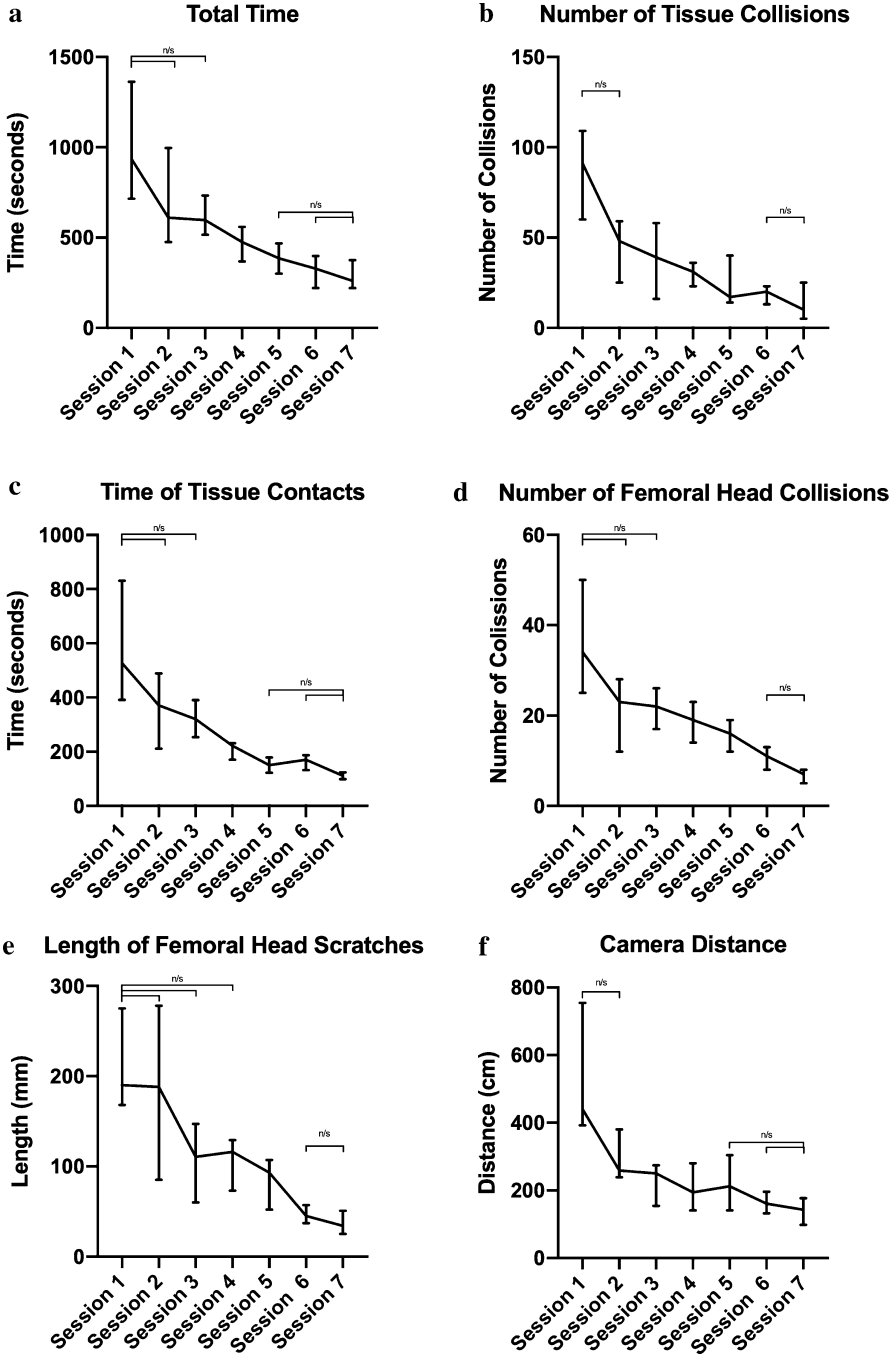
Student performance on arthroscopic visualisation module. All data presented as median ± interquartile range. All comparisons between session 1 and all other sessions, and between session 7 and all other sessions are significant (*p* < 0.05) unless otherwise stated (n/s: not significant).** a** Total time. Total time taken for student to complete module.** b** Number of tissue collisions. Number of collisions between the arthroscope and soft-tissue during the module.** c**Time of tissue contacts. Total time the arthroscope was in contact with soft-tissue during the module.** d** Number of femoral head collisions. Number of collisions between the arthroscope and the femoral head during the module.** e** Length of femoral head scratches. Total length of scratch on the femoral head caused by collisions with the arthroscope.** f** Camera distance. Total distance travelled by arthroscope during the module

Learning curve were demonstrated by the students, with significant improvements in all parameters following training (Fig. 2). Average total time decreased by nearly 75% across the course—936 s (657–1688) to 261 s (207–379) (*p* < 0.0001) (Table 2). The first significant increase from the participants baseline was seen in session 3 (*p* = 0.024) and no significant difference from session 7 (peak performance) was first found at session 5.

**Table 2:** Median student performance in all parameters with Friedmann’s multiple comparisons test

	Session 1	Session 2	Session 3	Session 4	Session 5	Session 6	Session 7
Total time (s)	936 (657–1688)	610 (421–1014)	597 (445–850)	476 (318–702)	386 (229–599)	328 (219–432)	261 (207–379)
vs. Session 1	–	*p* = 0.3458	*p* = 0.0238	*p* < 0.0001	*p* < 0.0001	*p* < 0.0001	*p* < 0.0001
vs Session 7	*p* < 0.0001	*p* < 0.0001	*p* < 0.0001	*p* = 0.0024	*p* = 0.1317	*p* > 0.9999	–
Number of soft-tissue collisions	91 (44–126)	48 (23–66)	39 (16–59)	31 (18–39)	17 (14–44)	20 (13–28)	10 (5–27)
vs. Session 1	–	*p* = 0.6524	*p* = 0.0126	*p* = 0.0009	*p* < 0.0001	*p* < 0.0001	*p* < 0.0001
vs Session 7	*p* < 0.0001	*p* < 0.0001	*p* < 0.0001	*p* = 0.0002	*p* = 0.0064	*p* = 0.5703	–
Number of bony collisions	34 (25–79)	23 (10–29)	22 (13–27)	19 (11–25)	16 (9–21)	11 (8–16)	7 (4–9)
vs. Session 1	–	*p* = 0.1012	*p* = 0.0845	*p* = 0.0007	*p* < 0.0001	*p* < 0.0001	*p* < 0.0001
vs Session 7	*p* < 0.0001	*p* < 0.0001	*p* < 0.0001	*p* = 0.0024	*p* = 0.0324	*p* = 0.6524	–
Total time of soft-tissue collisions (s)	527 (369–994)	371 (185–500)	320 (232–442)	222 (153–231)	150 (98–266)	170 (123–187)	111 (94–144)
vs. Session 1	–	*p* = 0.6969	*p* = 0.2002	*p* = 0.0003	*p* < 0.0001	*p* < 0.0001	*p* < 0.0001
vs Session 7	*p* < 0.0001	*p* < 0.0001	*p* < 0.0001	*p* = 0.0024	*p* = 0.1207	*p* > 0.2972	–
Total length of femoral head scratches (mm)	190 (120–285)	188 (68–325)	111 (47–163)	131 (44–131)	93 (44–121)	45 (33–60)	34 (22–85)
vs. Session 1	–	*p* > 0.9999	*p* = 0.1106	*p* = 0.0845	*p* = 0.0050	*p* < 0.0001	*p* < 0.0001
vs Session 7	*p* < 0.0001	*p* < 0.0001	*p* < 0.0001	*p* = 0.0002	*p* = 0.0064	*p* > 0.9999	–
Distance travelled by arthroscope (cm)	440 (355–1156)	259 (214–397)	250 (129–347)	194 (140–293)	212 (101–346)	161 (118–221)	143 (67–212)
vs. Session 1	–	*p* = 0.1106	*p* < 0.0001	*p* < 0.0001	*p* < 0.0001	*p* < 0.0001	*p* < 0.0001
vs Session 7	*p* < 0.0001	*p* < 0.0001	*p* = 0.0101	*p* = 0.0080	*p* = 0.0101	*p* > 0.9999	–

Collisions between the arthroscope and soft-tissues decreased across the sessions by nearly 90%—91 (44–126) to 10 (5–27) (*p* < 0.0001). The first significant improvement from baseline was found at session 3 (*p* = 0.013) with no significant differences from session 7 found at session 6. Similarly, collisions between the femoral head and the arthroscope also decreased—34 (25–79) to 7 (4–9) (*p* < 0.0001). These improvements were first seen in session 4 (*p* = 0.0007) and no significant difference from session 7 was first found at session 6.

Total time of soft-tissue collision was found to decrease from 527 s (369–994) to 111 s (94–144) (*p* < 0.0001), with the first significant decrease seen in session 4 (*p* = 0.0003) and no significant difference from session 7 was first found in session 5. Length of femoral head scratches showed similar decreases—190 mm (120–285) to 34 mm (22–57) (*p* < 0.0001), with the first significant decrease in session 5 (*p* = 0.005) and no significant difference from session 7 first found in session 6.

Participant’s efficiency of movement was also found to improve, with total camera distance reaching 143 cm (67–212) from an initial 440 cm (355–1156) (*p* < 0.0001). The first significant increase was seen in session 3 and no significant difference from session 7 was first found in session 6.

## Discussion

The most important finding of the present study was the demonstration a learning curve for a visualisation module on a previously validated hip arthroscopy simulator [15]. Significant increases were seen in six simulator measured parameters after a minimum of three sessions, before a plateau was reached after a maximum of six sessions. These results mimic those of similar studies into the learning curves associated with virtual reality orthopaedic simulators, and support its potential use in the development of basic hip arthroscopic skills such as visualisation [13, 18–24]. The assessment of learning curves, or ‘training effects’ is an essential aspect of VR simulator validation. It demonstrates that the VR environment generated by the simulator has sufficient visuo-haptic consistency, enabling individuals to develop psychomotor skills within it. Without demonstration of this training effect, it cannot be assumed that the visuo-haptic feedback provided by the VR simulator is effective in enabling dextrous development.

Previous studies have demonstrated learning curves and training effects for other arthroscopic simulators. Pollard et al. demonstrated a learning curve for lateral and supine patient positions in simulated hip arthroscopy using a box simulator, showing improvements in time taken and efficiency of movement [13]. Similar learning curves have been demonstrated using the Sheffield Knee Arthroscopy Training System and a passive haptic knee arthroscopy simulator with medical students [19]. A particularly difficult learning curve for knee arthroscopy was noted in a similar study by Rahm et al. using a passive haptic knee arthroscopy simulator [23]. Additionally, a learning curve has been demonstrated for the insightMIST (GMV, Madrid, Spain) shoulder VR simulator, further supporting shoulder VR simulators’ implementation in surgical training [20]. Furthermore, the retention of the skills acquired during simulation has been investigated by monitoring performance over a more protracted period, demonstrating limited degradation over time [18, 21].

A small number of studies have assessed the real-world benefits of VR simulators, showing improved performance in an operative setting following simulation training [5, 11]. Cannon et al. showed orthopaedic residents randomised to receive VR simulator training outperformed their control group counterparts in an operating theatre [25]. These benefits to knee arthroscopy were also assessed by Waterman et al. who measured the improvements to performance, and compared them to another group trained on cadaveric specimens [26]. Similarly, Banaszek et al.’s study assessed improvements in arthroscopic performance in medical students trained on a VR knee arthroscopy simulator, demonstrating significantly greater performance when compared to controls [27]. Contrary to these promising results, the benefits of knee arthroscopy simulation were not found to be significant in a study by Rebolledo et al. when testing orthopaedic residents’ performances on cadaveric models after simulation training [28]. However, this study did find significant improvements in shoulder arthroscopy performance compared to controls.

### Limitations

Although the demonstration of a learning curve for this simulator is reassuring, it does not quantify the real-world benefits of training with this simulator—concurrent validity. Further studies are, therefore, required to confirm and quantify the concurrent validity of this simulator. This additional information will better inform orthopaedic training programmes as to the effectiveness of simulation training, and, as to when in a trainee’s career it should be utilised for the greatest benefit.

Additionally, this study is unable to provide insight into the potential cost benefits of simulator training, something particularly important given the high initial cost of purchasing a VR simulator. The ‘Transfer Effectiveness Ratio’ (TER), the only validated measure of cost effectiveness, is a tool commonly used in the aviation industry to quantify the difference in time required to achieve fully competent performance between virtual reality and real-life, with a ratio of 0.50 indicating that 1 h of simulator training saves approximately 30 min of operative training time [29, 30]. Implementation of such a measure in the future assessment of orthopaedic virtual reality simulators would allow both direct comparison between validated simulators and more accurate calculation of cost savings of simulator training.

This study has only demonstrated the learning curves for one of this simulator’s modules and one arthroscopic skill—‘scope manipulation’. This simulator contains numerous modules for hip arthroscopy, including probing modules, complex pathological cases, and shoulder and knee arthroscopy set-ups. Our results only show the learning curve of the module utilised and any conclusions drawn cannot be extended to the other modules of this simulator or other skills, for example, probing.

In addition to this, the rapid improvements displayed in this study are far quicker than the measurable improvements found in real-world hip arthroscopy learning curves. A recent systematic review suggested that after 30 cases significant reductions in operative time and complication rates are seen with surgeons training in hip arthroscopy [3]. Although the shorter learning curve found with this VR simulator suggests that VR simulation lacks the heterogeneous nature of arthroscopic cases seen in the real world, this is due to the role of the modules tested in training basic arthroscopic skills, such as camera manipulation and triangulation—they are not intended to mimic the complexity of real-world pathological cases. Furthermore, though a plateau was demonstrated after six sessions, this conclusion is limited as it was found toward the end of the testing period. As such further improvements may have been detected if additional sessions were tested.

## Conclusions

The results of this study demonstrate learning curves for a hip arthroscopy simulator, with significant improvements seen after three sessions. All performance metrics were found to improved, demonstrating sufficient visuo-haptic consistency within the virtual environment, enabling individuals to develop basic arthroscopic skills.
